# Reorienting Collaborative–Creative Methodologies: Reflexive Devices in the Interplay of Ethnographic and Mobility Research

**DOI:** 10.1177/10778004251412872

**Published:** 2026-01-15

**Authors:** Silvia Almenara-Niebla, Kevin Smets, Amanda Alencar

**Affiliations:** 1 Universidad Autónoma de Madrid, Spain; 2 Vrije Universiteit Brussel, Belgium; 3 Erasmus University Rotterdam, The Netherlands

**Keywords:** creative-collaborative methods, reflexive devices, mobility and immobility, ethnography, ethnographies, methodologies, participatory research

## Abstract

This Special Issue engages current debates on creative–collaborative methods (CCMs) as a means of reconfiguring qualitative knowledge production beyond conventional hierarchies. Bringing together arts-based, participatory, community-oriented, and digitally mediated approaches, the contributions foreground collaboration as an ethical and methodological orientation within ethnographic research on mobility. Central to the issue is the notion of reflexive devices: situated field practices that operate simultaneously as methodological tools and ethical propositions. These devices foreground care, reciprocity, and accountability across conditions of (im)mobility. Collectively, the articles reimagine ethnography as a reflexive, relational process through which methodological innovation emerges from epistemic responsibility.

## Introduction

This Special Issue of *Qualitative Inquiry* joins a current development in qualitative research to engage more profoundly with creative–collaborative methods (CCMs). We use CCM as an umbrella term for scientific practices that approach knowledge production together with participants in ways that go beyond traditional qualitative methods. This includes a wide range of techniques such as arts-based methods, participatory action research (PAR), community-based research, and research using digital technologies, whereby participants’ experiences and perspectives are foregrounded. The dynamism around CCM is exhilarating, following landmark handbooks (e.g., Jason & Glenwick, 2016; Kara, 2015; Leavy, 2009; McIntyre, 2008) that have embraced community-oriented qualitative research that bridges scholarly and artistic practices. There have recently been numerous forums for these approaches, including volumes and special issues in qualitative methodology (Burns et al., 2021; Gerber & Siegesmund, 2022; Schubotz, 2019) and scholarly calls for more global collaboration and networking around such approaches (Gerber et al., 2020). This dynamism did not come out of the blue. In various disciplines, (visual) arts, creative forms of data collection, analysis and dissemination, and/or collaborative approaches to knowledge development can be traced back far beyond the current growth of publications and projects. Aspects of CCMs have been well documented in fields as diverse as anthropology, health and medicine research, pedagogy, and design research. In many of these cases, CCMs have been adapted to the framework of ethnography, especially after the profound reflections within ethnographic practice on the co-production of knowledge (Clifford & Marcus, 1986). Historically however, CCM approaches have often been situated more at the margins of those fields, partly due to debates around colonial epistemologies (in visual anthropology, see Edwards, 1992), reliability and validity (in arts-based research with small samples, see Siegesmund, 2014), and the emancipatory potentials of CCMs (e.g., in participatory research, see Campbell, 2002).

So, what is new then, about CCMs and our current timeframe? We argue that the recent dynamism around these approaches stems from at least three larger developments. The first and most cynical concerns the neoliberal academic environment in which most researchers operate. Neoliberal universities tend to collaborate with organizations and communities outside the university partly as a strategy to move closer to the market (see Brackmann, 2015). And as these same universities compete for scarce resources and attention, science communication and community outreach risk becoming mobilized as part of such market logics. A great challenge for CCM is to resist this instrumentalization. A second development, perhaps the flipside of the coin, is the meaningfulness of CCM for researchers and participants. In neoliberal university environments where polycrisis and planetary urgencies are felt in their own ways (from publication pressures to budget cuts, from online teaching to academic burnouts and populist attacks on science), an increasing number of researchers find meaning and hope in reflexive and creative methods that acknowledge lived experience and positionality. CCM, with its emphasis on “lived knowledge,” promises gateways to break these barriers down (Higgins & Lenette, 2024). A third and more pragmatic development is the larger toolbox that CCM can now choose from. Digital technologies, while not a guarantee for more inclusion and creativity *per se*, have been enthusiastically embraced by qualitative researchers to develop more horizontal engagements with participants or to develop co-creative strategies (Sarria-Sanz et al., 2024).

Within CCM research, particularly in decolonial and feminist frameworks, there is also a growing emphasis on “empowering” marginalized communities. Although empowerment is a complex and contested concept, it generally entails increasing individuals’ authority and enabling them to attain greater power (Aziz et al., 2011). This process allows them to exercise agency in decision-making, particularly in articulating their views, aspirations, and needs during the research process. Lenette (2022) argues for the importance of centralizing the knowledge and perspectives of marginalized groups as an “effective decolonial tool” for addressing the academic, political, economic, cultural, and social barriers that create and perpetuate systems of inequality and violence. Collaborative approaches indeed offer meaningful opportunities to promote inclusive research practices and decolonial values (Held, 2019), while fostering subjective, affective, cultural, and creative ways of knowing that can challenge Western academic standards regarding what is deemed acceptable or “valid” forms of knowledge (see Fiddian-Qasmiyeh, 2024).

However, engaging in “meaningful collaboration” within CCM also requires a critical examination of how the concept of agency is understood and experienced across various research phases and contexts, especially in relation to traditional narratives that often exaggerate agency and empowerment in collaborative and creative research (Cook et al., 2019). Simply asserting a commitment to collaboration does not guarantee the adoption of approaches that facilitate negotiations of power imbalances; instead, such collaborations may require navigating instances of “given” and “taken” agency. This understanding is further enriched by considerations of positionality in feminist and decolonial research, where ethics of care become paramount (Nasser-Eddin & Abu-Assab, 2020). Critiques of collaborative methods in this context have centered on the failure to incorporate discussions of intersectional factors and positionality, which are essential for fostering agency and democratizing knowledge production in the research process.

CCM approaches have recently garnered significant attention from ethnographers seeking to reconceptualize collaboration in research through a decolonial critique of the relational dynamics inherent in fieldwork. As Marcus (2018) suggests, collaboration is related to the change of old practices and habits of relation in social research with the aim of rethinking the role of the researcher and their impact on the ethnographic field; collaboration “has the potential of altering older habits and practices of achieving” (p. xi), implying new orientations on ways of doing research. This Special Issue examines the methodological innovations that CCM has introduced to ethnographic practice, with particular emphasis on their implications for mobility studies. Specifically, it explores how CCM contributes to reconfiguring hierarchical research structures while integrating media practices as a means of fostering more equitable knowledge production. As will be discussed in the following sections, this reconfiguration has profoundly influenced the reflexive practices of ethnographic research, particularly in its intersection with media.

## Creative–Collaborative Methods and Ethnography

As a result of the broader epistemological and methodological shifts outlined above, current debates in ethnography are increasingly oriented toward forms of research that not only describe the present but also aspire to generate change and imagine alternative futures. This orientation, as Bryant and Knight (2019) argue, can be understood through the cycle of plan, hope, and imagination, which frames ethnographic practice as inherently future-oriented. Similarly, Ahmed’s (2006) reflections on orientation emphasize how research trajectories are shaped by affective investments and directional choices that position researchers and participants toward certain directions. These directions make reflexivity indispensable: ethnographers must continually interrogate how collaborative engagements and creative practices produce knowledge. In this sense, CCMs exemplify an ethnographic orientation that is at once reflexive and aspirational: they foreground the relational dynamics of research while opening space for alternative ways of knowing and being in the field.

In recent decades, ethnography has been characterized by three interrelated advances: the rise of experimental and reflexive practices (Almenara-Niebla & Smets, 2025), the redefinition of “the field” and its temporality (Pink, 2018), and the methodological expansion toward multisite approaches (Marcus, 1995). As Pink (2018) points out, the ethnographic process is constituted, shared, and invented through relationships with others, which are formed through collaboration and experimentation. From her perspective, it is now necessary to reconsider what constitutes the field of ethnography and its temporality. In a context driven by globalization and mobility, classical conceptions of ethnography no longer fit, as spending long periods of time in the field to gain an insider’s perspective does not allow one to follow the objects, people, dynamics, and processes of the moment (Marcus, 1995). Instead, in a mobile world, ethnography functions as a “mixed practice” (Pink, 2018, p. 202), encompassing approaches that transcend traditional disciplinary boundaries between theory and practice.

Following these questions, CCMs have become increasingly relevant in this context. They destabilize conventional ethnography and align with the transformations described above. First, CCMs foster the co-creation of knowledge through participatory and creative practices, expanding reflexive ethnographic traditions while redistributing authority between researchers and participants (Lassiter, 2005). Second, CCMs introduce new modes of engagement that unsettle the classical model of long-term immersion, emphasizing relational, iterative, and often digitally mediated connections (Postill, 2011). Third, in multisited research, CCM expands the methodological repertoire by following stories, people, objects, and events across spaces and creative platforms, such as participatory video, art, or performance (Jokela-Pansini, 2019).

In this regard, there is a fundamental questioning of traditional ethnographic methods, such as participant observation, which are also being reconfigured through CCM. Once understood as privileging the researcher’s insider–outsider perspective, observation can now be framed as a collaborative and improvisational process shaped by the presence and agency of participants (Arribas Lozano, 2022). CCMs enable polyvocal knowledge production, where co-writing, storytelling, and arts-based collaboration allow multiple perspectives to shape the ethnographic record (Nikielska-Sekula & Desille, 2021). They also foster new ethical terrains by redistributing authority and inviting shared decision-making; CCMs challenge extractive research practices and generate accountability to participants’ needs and aspirations. Moreover, CCMs expand the temporal and spatial reach of ethnography. Iterative engagements, digital connections, and long-term collaborations allow fieldwork to exceed the bounded temporality of traditional approaches, capturing dispersed, transnational, and dynamic phenomena. Creative outputs—such as exhibitions, performances, or digital archives—extend the impact of ethnography beyond academia (Martínez, 2023). In this sense, CCMs amplify the transformative potential of ethnography, positioning it not only as a mode of research but as a collaborative practice of knowledge production.

The concept of field devices is central to understanding how CCMs reshape ethnography. Following Estalella and Criado (2023), analysis and methods are deeply intertwined with the field, encompassing both the physical and virtual spaces in which research occurs. These devices are embedded in the social realities of participants, capturing situated knowledge while fostering reflexivity and collaboration. As Malkki (2007) notes, “fieldwork is an embodied and embodying form of knowledge production” (p. 176), relying on the active participation of others. Today, following these experiences, ethnography also appears to be facing new challenges related to the inclusion of CCM. Although recent literature has reviewed these challenges and has begun to question certain traditional research practices (Criado & Estalella, 2018; Pink, 2018), many advocates of creative methods offer limited or no analysis of the role of ethnography in developing, implementing, and consolidating its creative and collaborative practices. Most of these studies do not reflect on the principles of ethnography and participant observation in the implementation of collaborative or creative methods (Kara, 2015). However, ethnographic experiences and the consolidation of a specific field are crucial to develop more participant-engaged practices, as well as to promote less hierarchical research approaches.

These challenges and opportunities are amplified in the context of a mobile, globalized world. The long-term reconfiguration of fieldwork methods and the move beyond traditional approaches to researcher–participant interactions have opened up new possibilities for exploring the field. However, the challenges associated with a dynamic and interconnected global landscape, alongside the limitations of methods originally developed to study small-scale societies, have been critiqued as inadequate for addressing contemporary social issues related to mobility (Gupta & Ferguson, 1997). Thus, the reconfiguration of broader fieldwork approaches also fitted into the methodological response needed to face the challenges linked to globalization (Appadurai, 1996).

In this sense, CCMs emerge not only as methodological innovations but as essential tools for ethnography in a mobile world. They allow researchers to attend to relationality, temporality, and transience, generating knowledge collaboratively while responding to the ethical, affective, and participatory imperatives of contemporary fieldwork. This sets the stage for exploring mobility-specific research practices, where creative and collaborative methods provide the tools to understand movement as a central organizing principle of social life.

## Bridging Ethnography and CCM: The Mobility Turn

In relation to these challenges in methodological innovations in ethnography, the concept of mobility is key to understanding the transformation of the ways in which ethnographers understand social life, shifting attention from fixed places to flows, circulations, and relational processes. Foundational contributions by Cresswell (2006) and Sheller and Urry (2006) conceptualize mobility as socially, culturally, and politically produced, highlighting movement as central to understanding modern life. As previously mentioned, ethnography, traditionally rooted in extended observation at a single site, is increasingly challenged to account for these dynamics. In this context, CCMs offer a means to study mobility in ways that capture histories, map locations, and reconfigure processes within the variety of mobility experiences, especially related to migration and diasporic networks.

Research on mobility represents a particularly fertile area for building bridges between ethnography and CCMs, as it challenges methodological nationalism and the structural constraints imposed by capitalist political economy (Wimmer & Schiller, 2003). This body of research has provided an analytical framework for understanding how social and economic activities, as well as interpersonal relationships, are constituted within transnational social fields that extend beyond state borders (Basch et al., 1993). Recognizing the need to conceptualize social life as a dynamic process that transcends borders in multiple ways, studies on mobility have reframed migration as a continuous flow that interconnects spaces, individuals, and information.

Despite the central importance of mobility in contemporary ethnography, immobility has also received methodological or analytical attention. While movement has been theorized as a constitutive feature of global social life, immobility has often been framed in negative terms, associated with restriction, exclusion, or spatial confinement. The study of immobility has therefore been particularly prominent in ethnographies of refugee camps, border zones, and other “places of exception” (e.g., Diken & Laustsen, 2005). However, as recent research shows (Smets, 2019), experiences of stillness, waiting, or restriction can also reveal complex forms of agency, experiences, and relationships that remain unexplored in mobility-focused research. Therefore, paying attention to immobility as an ethnographic condition opens up new perspectives on how movement and stillness are intertwined in the production of social life.

Building on the relationship between mobility and immobility, these approaches extend ethnographic research beyond conventional fieldwork by embracing and reconfiguring different modes of knowledge construction (e.g., Falzon 2009; Marino, 2020; Pasura, 2022). In this sense, much of the literature categorized as “arts-based research” has narrowly focused on the methods themselves—such as creative writing or visual arts, including drawing, painting, collage, and photography (Kara, 2015)—without adequately valuing the “field” in which the research takes place. However, following Dréano and Rudolfi (2021), analysis and methods are deeply intertwined with what is often referred to as the “field.” This term encompasses the space where the research is conducted, involving fieldwork and participant observation in everyday life, whether physically or online. Many contributors to this special issue describe and contextualize CCM within the framework of a specific field that is defined and constructed through situated knowledge (Haraway, 1988). This knowledge emerges from collaboration with participants, an understanding of the context of mobility, and an appreciation of their social realities. These modes of knowledge shape the field in which the research unfolds. Furthermore, through the incorporation of different devices (Estalella & Criado, 2023), they establish a critical research approach, integrating collaborative practices.

The contributors to this special issue embody this adaptability, using CCM to consolidate transformative research practices that generate change and respond to evolving realities. The authors reformulate conventional participant observation through participatory, decolonial, action-oriented, digital, and arts-based approaches. The notion of field device has inspired the authors of this Special Issue. Moving beyond the vocabulary on creative or innovative methods, the research presented here tries to challenge and transform the field “from the bottom up by intervening at the site of its production” (Culhane, 2017, p. 7). The intervention in the field and its reexamination also represent not just a methodological concern, but also an ethical one. Most of these devices included during the ethnography or participant observation studies have emerged in a reflexive moment that actively understands how the production values of the research become socialized, aesthetic, and affect the moral sensibilities that shape the ethnographic encounter. This also implied that “field devices lack the stability and standardization attributed to methods” (Estalella & Criado, 2023, p. 2), which creates uncertainties and, indeed, reorientations in the research path. In that sense, we argue that CCMs are field devices that require certain sensitivities on the part of the researcher to break with classical research hierarchies.

According to Ballestero and Ross (2021), creative forms of thinking have brought together many inventive techniques inside ethnography to capture analysis. In this sense, following the notion of field device, we argue here that CCMs are not just methods or techniques that capture that inventiveness in terms of data collection during a particular moment of the research. We believe that the use of diverse field devices is inherently linked to reflexive practices as an integral part of the research process. This reflexive moment forms a crucial aspect of the research journey, where the researcher places significant value on the collaborative dimensions of the study through attentive listening and by giving greater prominence to the participants due to the influence of decolonial and feminist views on conducting research.

The authors of this Special Issue address the challenges of research in mobility contexts and emphasize the necessity of incorporating new approaches to engage with sensitive knowledge. Underpinning these efforts is a thoughtful reflection on the hierarchies inherent in research, highlighting the importance of care and equity in the research process. In that sense, CCM has opened up the discussion about the impact of intersectional positionality (Moralli, 2024), which delves into the position the researcher reaches at different moments of the research, where the emotions and sensitivities of the ethnographic encounter set the research agenda. The use of CCM-based field devices is fundamental here, as it will condition the researcher’s relationship with the participants, generating a greater focus on issues of care and collaboration.

## Reflexive Devices: Positionality, Multimodality, Adaptability, Intersectionality

As demonstrated in the articles in this Special Issue, the researchers adopted a critical stance toward their research agendas, centering the participants and co-researchers within a framework of care. The choice of one method over another, and often a combination of various approaches, lies in their attentiveness to power dynamics, hierarchies, and the necessity of addressing these differences throughout the research process. Consequently, we argue that the use of a specific field device is not solely dictated by the conceptual demands of the research or the need for creative adaptation to the field’s requirements. Rather, it is deeply connected—both reflexively and practically—to the participants and the relational commitments established with them. This integration results in what we term a reflexive field device, grounded in care and mutual engagement. By weaving together these reflexive field devices specific to each case, authors reconsider practices and modes of knowledge throughout the research process, encouraging debate at the intersection of ethnography and mobility. Four specific devices that are strongly developed in this Special Issue: positionality, multimodality, flexibility, and intersectionality.

*Positionality*, as the primary reflexive device, refers to the perspectives and viewpoints of a researcher during the research process. It outlines the researcher’s stance in relation to the way in which the study is carried out, influencing theoretical, methodological, and empirical decisions (Rowe, 2014). It also emphasizes how researchers view themselves and how others perceive them, whether as insiders or outsiders, or as individuals with privilege or in a disadvantaged position. Multiple factors (to be discussed in *Intersectionality* later), such as gender, race, experiences, values, and beliefs, shape positionality, as well as assumptions, research interactions, and approaches to data engagement. In this regard, scholars argue that positionality is not always linear or defined by simple binary relationships of insider/outsider; rather, it is fluid, open, formed along a continuum, and deeply contextual (Eppley, 2006). Building on ethnography, Jo Warin (2011) argues that by engaging in reflexivity and maintaining ethical mindfulness, researchers can critically evaluate the knowledge they generate and reflect on how their experiences and circumstances impact the research process and its results. More broadly, scholars using qualitative methods argue that practicing reflexivity through positionality improves the trustworthiness and validity of findings, as it helps researchers better understand their own values, biases, and beliefs (Yip, 2023). In participatory research approaches, acknowledging their own privileges and questioning their prejudices enables researchers to build reciprocal relationships with “co-researchers” that are crucial for sustaining ethical, honest, and respectful collaborations (Lenette, 2022, p. 9).

CCM can significantly benefit from incorporating positionality, as this reflexive tool provides an opportunity to address power imbalances that hinder meaningful collaborations between researchers and participants. This, in turn, promotes equitable partnerships, skill development, and shared ownership in projects. While it does not mean that power imbalances will disappear, positionality can help foster equitable research participation by recognizing power differentials and creating culturally safe spaces for knowledge exchange. The authors of articles in this Special Issue highlight the importance of discussing their positionality as a means to foster reflexivity across the research process. This emphasis enriches the development of creative–collaborative approaches in ethnographic fieldwork.

*Multimodality* comes in as a second important reflexive device. Multimodality refers to an awareness of how multiple modes of communication (text, image, sound, gesture, artifacts) produce and interpret meanings. The core idea is that meaning-making is not confined to one of those modes, but instead shaped through their interplay, and always composed of many parts. These ideas are rooted in a long tradition of social semiotics (Halliday, 1978) and have particularly solidified in communication, discourse, and linguistics research (Kress & van Leeuwen, 1996; Machin, 2013). For social scientists, notably ethnographers and those doing different forms of fieldwork, multimodality is a useful focus too. While attention to non-linguistic modes of meaning-making (e.g., through materials, objects, images, sounds) has a very long tradition in ethnography (Dicks et al., 2011: 230), multimodality holds particular relevance in the contemporary timeframe. In the context of anthropology, Dattatreyan and Marrero-Guillamón (2019) stress that multimodality refers both to the media-rich worlds we inhabit (multiple media) and to the multisensorial engagements with the field (multiple senses). This perspective reshapes anthropology, and we would argue social sciences more broadly, by being more attentive to “the diverse ways of knowing the human experience” (Westmoreland, 2022: 174).

The authors in this Special Issue do not necessarily adopt a multimodal framework, nor do they identify as ethnographers in the strict sense (for more specific, in-depth reflections on the intersections between multimodality and ethnography, see the special issue of *Qualitative Research* edited by Dicks et al., 2011). But awareness of multimodality is woven into much of the work that we have gathered here, and looking through that lens even briefly demonstrates how it can enrich the analytical scope of CCM. The studies in this Special Issue show, for instance, that a multimodal participatory design may multiply the possibilities for genuine participation simply by offering a broader set of instruments to communicate and express.

*Flexibility*, from a methodological point of view, aligns very much with this multimodal awareness. Attitudes of openness and adaptability have long been discussed as a key part of fieldwork, which has an inherently “messy” character. Iteration and nonlinearity are also widely seen as key dimensions of qualitative research. As a reflexive device in CCM, flexibility is a particular necessity to develop genuine participation that foregrounds lived experiences. Flexibility is needed to adjust to unexpected context developments, to navigate complex institutional structures, and to accommodate the ever-changing relationship with participants (CohenMiller et al., 2020; Cornish et al., 2023). Moreover, flexibility and a constant search for new pathways to produce and disseminate knowledge are essentially built into creative and arts-based research. Flexibility also means avoiding determinism in the CCM research design. In different ways, the papers in this special issue illustrate how they put flexible-reflexivity into practice.

### Intersectionality

The increasing use of CCM methods, like participatory, decolonial, action-oriented, digital, and art-based approaches, highlights the role of intersectionality as a crucial reflexive tool/device that addresses ethical considerations in research. In feminist research, intersectionality serves as a key theoretical and methodological framework for examining issues of exclusion, identity, and oppression (Lenette, 2022). In this context, Leslie McCall (2005) has noted that intersectionality theory is “the most important contribution that women’s studies and feminism have made so far” (p. 1771). Beyond academia, intersectionality also impacts policy, advocacy, and practice in the realms of human rights and social justice (Yuval-Davis, 2006). An intersectional approach focuses on recognizing the diverse experiences of individuals and how multiple aspects of their identity can increase or decrease their vulnerability to oppression (Carastathis, 2014). In tracing the roots of the concept of intersectionality, scholars refer to the important contribution of Black women, Chicana, and Latina women in mobilizing the term politically. However, the metaphor of “intersecting categories of discrimination” was introduced by American legal scholar Kimberlé Williams Crenshaw (1991) to highlight how traditional definitions of discrimination fail to adequately address and repair the experiences of discrimination faced by Black women at the intersection of both race and gender. Stated differently, it is impossible to comprehend Black women’s experiences without considering gender concerns in addition to other markers of oppression and identity, like race, class, disability, education, age, visa status, nationality, religion, sexual orientation, and cultural backgrounds. Since then, the term has gained significant traction in academic literature as a paradigm for exploring identity and power relations across various disciplines (for an overview, see Carastathis, 2014).

In this Special Issue, the contributions illustrate that using an “intersectional ethical device” with CCM methods can help contextualize the experiences of both researchers and participants in relation to systems and structures of oppression within their research practices. Building on Lenette’s (2022) work, we contend that an intersectional reflexive device serves as a guide for researchers and participants to engage in open discussions about power dynamics, inclusive research practices, and the consequences of research in collaborative and creative settings.

## The Special Issue and the Reflexive Devices Involved

Throughout this Special Issue, contributors collectively mobilize reflexive devices that reimagine how migration, mobility, and the production of ethnographic knowledge can be approached from a methodological and ethical perspective. In the various contributions, mobility is not only a research topic but also a way of doing and thinking about ethnography, an invitation to move with participants, across disciplines, and through multiple sites of knowledge and representation.

Positionality runs through all these works as a dynamic and situated practice, rather than as a static reflection on identity. For example, Nikielska-Sekuła’s proposal on *multisensory positionality* broadens the conventional conception of the researcher’s point of view by incorporating the body and the senses into the analytical framework. This sensory and intersectional attention to embodiment underscores how knowledge is co-produced through affective encounters that unfold in motion. In particular, the article by Sommier and colleagues (in this issue) employs positionality as a tool to examine racial issues related to urban environments and their residents, highlighting how knowledge production practices often render the positionality of Whiteness in academia invisible. By employing datawalks—mobile methodologies that encourage collaboration between researchers and participants—the authors contend that White researchers frequently neglect to address their positionality or conceal their experiences when working with White participants, thereby reinforcing the prevailing discourse of Whiteness in academic contexts. Their research asserts that urban spaces can serve as arenas for the interaction between race and one’s positionality, providing a critical perspective on reflexivity that emphasizes action over mere inquiry. Similarly, in the work of Irene Gutiérrez Torres et al., the five authors view their positionality as a basis for exploring empirical examples that shed light on creative practices and the difficulties of ensuring meaningful participation from co-researchers in co-dissemination. Their reflections indicate how the privileges of academics are utilized in creative collaborative approaches to enhance lived experience-led knowledge and address epistemic injustice through co-writing opportunities, which serve as significant forms of co-dissemination in participatory research.

The Special Issue also demonstrates how multimodality functions as both an epistemological and creative reflexive device that expands the scope of ethnographic research. Marchevska and Defrin’s exploration of participatory filmmaking and Su and Valiquette’s adaptation of Instagram as a method highlight how digital and audiovisual forms open up plural sensory, aesthetic, and narrative registers for addressing migrants’ experiences. Through film and digital images, participants become mobile authors of meaning, bringing everyday gestures and creative agency to the fore as forms of ethnographic expression. Godin and Doná’s work delves deeper into this multimodal and mobile ethnography by demonstrating how photography, video, and exhibition can function as analytical and political tools in border zones. Through projects such as *Displaces* and *Reversing the Gaze*, their article reclaims the border as a visual and epistemic place of encounter, where ethnography itself becomes itinerant, crossing artistic, disciplinary, and geopolitical boundaries. These creative and collaborative methodologies embody multimodality as a form of movement and resistance, generating counter-visualities that challenge dominant representations of migration and border control. In particular, Su and Valiquette deliberately combine video *and* photovoice—offering a range of communicative modes such as written captions, music, moving and still images, hashtags—to encourage free-thinking and fuller expression among participants.

Adaptability emerges in these articles as a methodological and ethical response to the changing terrains of collaborative and mobile research. Medrado and Rega’s concept of *pluriversal mobilities* explicitly resists universalising tendencies by embracing the circulation of people, artifacts, and ideas across disciplines and epistemic traditions. This flexibility is also epistemological, highlighting how knowledge and practice travel, mutate, and coexist across South-South circuits. Similarly, Gutiérrez-Torres et al.’s framework of co-dissemination extends the participatory spirit of ethnography beyond fieldwork, demanding adaptive and context-specific practices that ensure the meaningful participation of forced migrants throughout the research process. They highlight that co-researchers from disadvantaged and marginalized groups encounter obstacles in sharing knowledge due to mobility restrictions, technological constraints, and security risks, despite the presence of institutional support, adequate funding, and thoughtful planning. Their emphasis on presence, accountability, reach, and return reframes dissemination as an ongoing, ethical, and situated practice rather than an endpoint.

Finally, intersectionality is woven throughout all contributions as a lens that reveals the interdependence of identities, structures, and practices. Su and Valiquette’s intersectional analysis of sexuality, gender, race, class, and nationality in digital participation dialogues with Nikielska-Sekuła and Sommier et al.’s efforts to situate embodied and racialized positions within broader systems of power. At the same time, the creative collaborations of Marchevska and Defrin, on the one hand, and Godin and Doná, on the other, highlight the multiplicity of migrant subjectivities and solidarities that emerge in different places of mobility and encounter. Together, these articles redefine reflexivity not as self-conscious introspection, but as a collective, multimodal, and mobile ethnographic practice that recognizes the porosity of research boundaries, the coexistence of multiple worlds, and the ethical imperatives of researching differently and in motion.

## Conclusion

This Special Issue emerged out of multiple scholarly, artistic, and societal developments that necessitate a deeper look into the potentials of CCM. While CCMs represent a highly diverse field of approaches, we have argued in this introduction that they are particularly beneficial to strengthen current research into mobility. Ethnographic research offers a fruitful basis for this exploration, as we have developed through the notion of reflexive devices. Taken together, the contributions to this Special Issue reveal that methodological innovation in mobility research emerges not from a search for novelty alone, but from an ethical and relational commitment to those who inhabit and co-construct the field. Each contribution demonstrates how reflexivity and care become methodological forces that shape the production of knowledge, guiding researchers to continually negotiate positionalities, responsibilities, inclusive participation, and power dynamics. The field devices presented here are not simply methodological interventions, but situated reflexive practices that make visible the entanglements of power hierarchies, responsibility, and care that constitute the research encounter. In doing so, the authors collectively reimagine ethnography as a space and a methodological process where reflexivity is central to the field encounter and its interactions.

Thus, the reflexive field devices discussed across the articles function as both methodological and ethical propositions in their intertwining with mobility studies. These reflexive devices compel us to rethink what it means to do research *with* rather than *on* participants, foregrounding processes of co-presence, reciprocity, and situated accountability in different (im)mobilities. These approaches highlight that fieldwork in contexts marked not only by movement but also by waiting, uncertainty, and enforced stillness cannot be disentangled from the relations of care and vulnerability that sustain it. Recognizing immobility as an ethnographic condition—whether imposed, chosen, or circumstantial—invites attention to the temporalities of waiting, the affective intensities of confinement, and the creative ways participants and researchers navigate constraints together. Rather than treating these as challenges to be overcome, the authors show how they can become productive sites of methodological creativity and epistemic renewal.

However, as mentioned above, structural forces such as neoliberal principles, funding logic, and the growing use of advanced digital technologies, including artificial intelligence (AI) developments, are shaping the way research is conducted. This could undermine the use of reflexive devices. We are well aware of the challenges this poses, especially in aspects related to knowledge construction and dissemination of research results. Aware of these issues, this Special Issue invites scholars to reconsider ethnographic practice as an open dialogue shaped by the contingencies of mobility, immobility, care, and encounter.
